# Neural computation with efficient population codes

**DOI:** 10.1186/1471-2202-14-S1-P129

**Published:** 2013-07-08

**Authors:** Brian J Fischer

**Affiliations:** 1 Department of Mathematics, Seattle University, Seattle, WA 92200, USA

## 

Brain function depends on populations of neurons that perform computations on perceptually and behaviorally relevant variables. One of the main goals of neuroscience is to understand how the responses of populations of neurons and the connectivity patterns between groups of neurons allow brains to perform a wide range of neural computations. The Neural Engineering Framework (NEF) is a promising approach to designing neural models that perform many neural computations [1,2]. The central thesis behind the NEF is that populations of neurons represent, and perform computations on, low-dimensional time-dependent variables. By characterizing how neurons in a population encode a variable, and how the variable can be decoded from the distributed representation, functioning neural circuits are constructed that allow a comparison with experimental data at a range of levels from single neuron responses to connectivity patterns to perceptual and behavioral performance. The particular models that result from this approach depend on how the neural encoding and decoding processes are characterized. This is where emerging principles of neural computation constrain models of neural encoding and decoding.

We describe how efficient codes are used to design neural circuit models that perform a wide variety of computations. The fundamental characteristic of the efficient code is that the neural representation is adapted to the statistics of the environment. Here, we take an efficient code to be one where the preferred stimuli are drawn from the prior distribution and the neural tuning curves are proportional to the likelihood function [3-6]. We show that in an efficient code, a general center-of-mass decoder can extract Bayesian estimates of encoded variables or functions of encoded variables, which allows for the construction of networks that perform many computations [3,4]. Networks constructed using the method we describe have several nice functional properties and match many experimental observations. First, neural tuning properties match the statistics of the variables they process. Second, in this framework, normalization is an essential computation at each stage of processing. This is consistent with normalization being described as a canonical neural computation [7]. Also, computation in networks of neurons using efficient coding is robust to neuronal loss and uses local connection rules. Finally, the networks we describe are flexible and can incorporate changes to environmental statistics or goals using gain modulation or changes in tuning curve widths. The overall result is to show the importance of several emerging principles of neural computation in an already successful modeling framework.
